# The Pretreatment Systemic Inflammatory Response is an Important Determinant of Poor Pathologic Response for Patients Undergoing Neoadjuvant Therapy for Rectal Cancer

**DOI:** 10.1245/s10434-016-5684-3

**Published:** 2016-11-21

**Authors:** Stephan B. Dreyer, Arfon G. M. T. Powell, Stephen T. McSorley, Ashita Waterston, James J. Going, Joanne Edwards, Donald C. McMillan, Paul G. Horgan

**Affiliations:** 1 Institute of Cancer Science, University of Glasgow, Glasgow, UK; 2 Institute of Cancer and Genetics, Cardiff University, Cardiff, UK; 3 Academic Unit of Surgery, School of Medicine, University of Glasgow, Glasgow, UK; 4 Department of Oncology, Beatson West of Scotland Cancer Centre, Glasgow, UK; 5 Section of Pathology, University of Glasgow, Glasgow, UK

## Abstract

**Background:**

Not all patients respond equally to neoadjuvant chemoradiotherapy (nCRT), with subsequent effects on survival. The systemic inflammatory response has been shown to predict long-term outcomes in colorectal cancer. The current study examined the association between systemic inflammation and nCRT in patients with rectal cancer.

**Methods:**

Between 1999 and 2010, patients who underwent nCRT were identified. Serum measurements of hemoglobin, C-reactive protein, albumin, modified Glasgow prognostic score (mGPS), and differential white cell counts were obtained before and after nCRT. The Rödel scoring system measured pathologic tumor regression, and magnetic resonance imaging and computed tomography determined radiologic staging.

**Results:**

The study included 79 patients. Of these patients, 37% were radiologically downstaged, and 44% were categorized as showing a good pathologic response (Rödel scores 3 and 4). As a validated measure of the systemic inflammatory response, mGPS (*P* = 0.022) was associated with a poor pathologic response to nCRT. A radiologic response was associated with a good pathologic response to treatment (*P* = 0.003). A binary logistic regression model identified mGPS (odds ratio [OR] 0.27; 95% confidence interval [CI] 0.07–0.96; *P* = 0.043) and radiologic response (OR 0.43; 95% CI 0.18–0.99; *P* = 0.048) as strong independent predictors of a pathologic response to treatment.

**Conclusion:**

The current study showed that a systemic inflammatory response before nCRT is associated with a poor pathologic response. Further study in a prospective controlled trial setting is warranted.

Colorectal cancer (CRC) is the third most common cancer and the second highest cause of cancer death in the United Kingdom.^1^ The 5-year survival rate for CRC still is less than 60% with surgery alone, offering the only chance of cure.

Rectal cancers comprise about one third of surgical resections for colorectal cancer.^2^ The widely adapted surgical technique of total mesorectal excision (TME), increased centralization, specialization of rectal surgery, and earlier disease detection have led to improved survival in the last 30 years.^3^,^4^ Preoperative neoadjuvant radiotherapy with or without chemotherapy currently is accepted as a standard of care for patients with margin-threatening rectal cancer. This increases disease-free survival (DFS) and sphincter preservation rates and improves circumferential resection margins and local recurrence rates.^5^–^8^

Current management of CRC in the United Kingdom involves evaluating patients using magnetic resonance imaging (MRI) and computed tomography (CT) before treatment to identify those with margin-threatening disease (T3 or T4).^9^ These patients are offered neoadjuvant chemoradiotherapy (nCRT) before surgical resection.^10^

Not all patients respond to nCRT, and there is a need to identify biomarkers of response because treatment is associated with significant morbidity. Rödel et al.^11^ have shown that the presence of spontaneous apoptosis in the resected specimen is a good marker of tumor regression and improved prognosis.

The prognostic value of the systemic inflammatory response (SIR) has been widely studied in gastrointestinal cancers, particularly in the operative setting, using measurements of circulating markers including C-reactive protein (CRP), albumin, the modified Glasgow prognostic score (mGPS), the neutrophil lympocyte ratio (NLR), the platelet-to-lymphocyte ratio (PLR), and more recently, the neutrophil-platelet score (NPS) and the derived neutrophil-to-lymphocyte ratio (dNLR).^12^–^16^

This study investigated the association between markers of the systemic inflammatory response and the pathologic response to nCRT in patients with rectal cancer.

## Methods

Patients who underwent nCRT and surgical resection for rectal adenocarcinoma between 1999 and 2010 were identified from a prospectively maintained colorectal cancer database. Patients with biopsy-confirmed adenocarcinoma of the rectum who received long-course nCRT and attempted curative resection were included in the study. Patients with margin-threatening disease and radiologic evidence of locally involved lymph nodes underwent nCRT as decided by the local colorectal cancer multidisciplinary teams. Patients were excluded from the study if they were considered unsuitable for long-course therapy, had undergone previous surgery for rectal cancer, or had documented chronic inflammatory conditions.

The patients were assessed and diagnosed according to standard national guidelines. Those with biopsy-confirmed rectal adenocarcinoma underwent staging CT and MRI to assess local extent of disease and evidence of metastases. These were repeated at the clinician’s request after nCRT for all the patients.

The nCRT regimen was at the discretion of the treating oncologists. Radiotherapy was delivered at a standard dose of 45 Gy in 25 fractions, with a range of 39.6–50 Gy. The concurrent chemotherapy regimens used included 5-fluorouracil (5-FU), capecitabine, or combinations of these with oxaliplatin and mitomycin.

## Clinicopathologic Characteristics

This study was approved by the West of Scotland Research Ethics Committee, Glasgow. Pathologic specimens were processed and reported by specialist gastrointestinal cancer pathologists, and final staging was reported as per the International Union Against Cancer (UICC) tumor-node-metastasis (TNM) staging system. This included a description of the lymph node ratio, the total lymph nodes retrieved, and tumor differentiation. Resection margin status was described as either free of tumor (R0), microscopically involved (R1), or macroscopically involved (R2).

Pretreatment blood test measurements were defined as the sample taken closest to the nCRT start date and included hemoglobin (Hb), neutrophil, lymphocyte and platelet counts, carcinoembryonic antigen (CEA), albumin, and CRP. Anemia was defined as levels lower than 13 g/dL (men) and lower than 11.5 g/dL (women). The CRP level was considered high if it exceeded 10 mg/L, and hypoalbuminemia was defined as a level lower than 35 g/L.

The mGPS was calculated as previously described.^1^ Briefly, patients with an elevated CRP were assigned an mGPS score of 1 or 2 depending on the presence or absence of hypoalbuminaemia. The NLR and PLR were calculated as standard, with cutoffs of 5 or lower (NLR) and 300 or lower (PLR) considered normal. The NPS was calculated as recently described.^15^ Patients with a neutrophil count lower than 7.5 × 10^9^/L and a platelet count lower than 400 × 10^9^/L scored 0. Those with either a neutrophil count higher than 7.5 × 10^9^/L or a platelet count higher than 400 × 10^9^/L scored 1, and those with both a neutrophil count higher than 7.5 × 10^9^/L and a platelet count higher than 400 × 10^9^/L scored 2. This measurement (the derived neutrophil-Lymphocyte ratio or dNLR) is calculated by the neutrophil count divided by the (white cell count minus the neutrophil count).^16^ The CEA was deemed normal at a value lower than 5 µg/L.

### Assessment of Response to nCRT

Radiologic response to therapy was defined as tumor downstaging according to pre- and postneoadjuvant treatment imaging. The MRI and CT images were reported by dedicated gastrointestinal (GI) radiologists with a special interest in colorectal cancer.

Specimen sections were used to determine the pathologic response to therapy by quantifying the tumor regression grade (TRG) as validated previously.^11^,^17^ Tumor regression was quantified by the relative amount of fibrosis compared with residual viable tumor, ranging from no response to no evidence of viable tumor remaining.^11^ Each individual grade was defined as follows: grade 0 (no regression), grade 1 (minor regression: <25% of fibrosis in the tumor mass), grade 2 (moderate regression: 26–50% fibrosis within the tumor mass), grade 3 (good regression: dominant feature of fibrosis [>50% fibrosis vs the tumor mass]), grade 4 (complete regression: no evidence of viable tumor mass).^11^ Patients were dichotomized as having a good response (TRG 3 and 4) or a poor or no response (TRG 0, 1, and 2).

### Statistical Analysis

Statistical evaluation was performed in SPSS (version 22.0; IBM SPSS Statistics, IBM Corp., Armonk, NY, USA). Grouping of the variables was performed using standard clinical thresholds. Comparisons between groups of patients were performed using contingency table analysis (Chi square) as appropriate, and Fisher’s exact test was used for those with *n* less than 5. Logistic regression was performed to determine univariate relationships between preoperative clinical predictors and response to nCRT. Multivariate logistic regression analysis, including all statistically significant covariates at a *P* value of 0.05 or lower, was performed by a stepwise backward procedure to derive a final model of the variables that had a statistically significant relationship between pathologic response and nCRT.

## Results

### Clinicopathologic Characteristics

The study identified 176 patients who underwent nCRT and surgical resection for rectal cancer in the period 1999–2010. Patients not suitable for long-course chemoradiotherapy (*n* = 66), those who did not complete the course of chemoradiotherapy (*n* = 4), those who had no pretreatment blood results available (*n* = 10), and those who had no postoperative histopathology samples available (*n* = 17) were excluded from the study, leaving 79 patients included for the analysis.

The majority of the patients were male (*n* = 56, 70.1%), and the median age of the studied cohort was 65 years (range 39–84 years). At initial presentation and radiologic examination, the majority of the patients had T3 disease (*n* = 50, 63.3%), 74.7% (*n* = 59) were node-negative, and 7.6% (*n* = 6) had evidence of metastatic disease. During the neoadjuvant treatment course, 37 of the patients (46.8%) were radiologically downstaged, 38% had no stage change, and 15% (*n* = 12) progressed radiologically (Table 1). Using the Rödel classification, tumors were classified as 0 (0%), 1 (22%), 2 (34%), 3 (41%), or 4 (4%) and thus dichotomized as poor or having no response (*n* = 44, 55.7%) or as having a good response (*n* = 35, 44.3%). At the final pathologic staging, 3 patients (3.8%) had a complete pathologic response (pT0), 4 patients (5.1%) were stage pT1, 13 patients (16.5%) were stage pT2, 47 patients (59.5%) were stage pT3, and 12 patients (15.2%) were stage pT4 (Table 2).

**Table 1:** Clinicopathologic characteristics of patients undergoing neoadjuvant chemoradiotherapy (nCRT) and surgery for rectal cancer

Factors	Patients (*n* = 79) *n* (%)
Sex	
Female	23 (29)
Male	56 (71)
Age (years)	
<65	36 (46)
65–74	34 (43)
>75	9 (11)
T-stage	
0 (complete pathologic response)	3 (4)
1	4 (5)
2	13 (16)
3	47 (60)
4	12 (15)
N-stage	
0	50 (63)
1	14 (18)
2	15 (19)
M-stage	
0	75 (95)
1	4 (5)
TNM stage	
0	3 (4)
1	13 (16)
2	33 (42)
3	26 (33)
4	4 (5)
Differentiation	
Moderately/well	68 (86)
Poor	10 (13)
Unknown	1 (1)
Vascular invasion	
No	48 (61)
Yes	30 (38)
Unknown	1 (1)
Perineural invasion	
No	67 (85)
Yes	9 (11)
Unknown	3 (4)
Resection margin	
Not involved	65 (82)
Involved	14 (18)
Neoadjuvant chemotherapy regimen	
Capecitabine	27 (34)
5-FU	44 (56)
Other	8 (10)
Surgical procedure	
Anterior resection	38 (48)
Abdominoperineal resection	30 (38)
Hartmann’s resection	2 (3)
Unknown	9 (11)
Radiologic response	
Good response	37 (47)
No response	30 (38)
Disease progression	12 (15)
Pathologic response	
No response	44 (56)
Good response	35 (44)

*TNM* tumor-node-metastasis, *5-FU* 5-fluorouracil

**Table 2:** Association between clinicopathologic factors and pathologic response to neoadjuvant chemoradiotherapy (nCRT)

Clinicopathologic factors	No response patients (*n* = 44) *n* (%)	Good response patients (*n* = 35) *n* (%)	*P* value
Sex			
Female	15 (34)	8 (23)	0.275
Male	29 (66)	27 (77)	
Age (years)			
<65	16 (36)	20 (57)	0.127
65–74	21 (48)	13 (37)	
>75	7 (16)	2 (6)	
T-stage			
0 (complete pathologic response)	0 (0)	3 (9)	<0.001^a^
1	0 (0)	4 (11)	
2	3 (7)	10 (29)	
3	31 (70)	16 (46)	
4	10 (23)	2 (6)	
N-stage			
0	24 (54)	26 (74)	0.189
1	10 (23)	4 (11)	
2	10 (23)	5 (14)	
M-stage			
0	41 (93)	34 (97)	0.399^a^
1	3 (7)	1 (3)	
TNM stage			
0	0 (0)	3 (0)	0.004
1	2 (4)	11 (31)	
2	22 (50)	11 (31)	
3	17 (39)	9 (26)	
4	3 (7)	1 (3)	
Differentiation			
Moderately/well	38 (86)	30 (88)	0.543^a^
Poor	6 (14)	4 (12)	
Vascular invasion			
No	19 (43)	29 (85)	<0.001
Yes	25(57)	5 (15)	
Perineural invasion			
No	36 (82)	31 (97)	0.045^a^
Yes	8 (18)	1 (3)	
Resection margin			
Not involved	34 (77)	31 (89)	0.156^a^
Involved	10 (23)	4 (11)	
Neoadjuvant chemotherapy regimen			
Capecitabine	16 (36)	11 (31)	0.875
5-FU	24 (55)	20 (57)	
Other	4 (9)	4 (11)	
Surgical procedure			
Anterior resection	21 (48)	17 (49)	0.911
Abdominoperineal resection	16 (36)	14 (40)	
Hartmann’s resection	1 (2)	1 (3)	
Unknown	6 (14)	3 (9)	
Radiologic response			
Disease progression	9 (21)	3 (8)	0.003
No change	22 (50)	8 (23)	
Good response	13 (29)	24 (69)	
mGPS			
0	21 (55)	22 (88)	0.022
1	9 (24)	1 (4)	
2	8 (21)	2 (8)	
Hemoglobin			
Normal (>13 g/dL [men]; >11.5 g/dL [women])	23 (53)	22 (65)	0.321
Anemia (<13 g/dL [men]; <11.5 g/dL [women])	20 (47)	12 (35)	
CEA			
Normal (≤5 µg/L)	14 (43)	18 (62)	0.152
High (>5 µg/L)	18 (56)	11 (38)	
NLR			
Normal (≤5)	33 (75)	32 (91)	0.052^a^
High (>5)	11 (25)	3 (9)	
dNLR			
Normal (<2)	22 (50)	19 (54)	0.705
High (≥2)	22 (50)	16 (46)	
NPS			
0	31 (70)	25 (71)	0.928
1	10 (23)	7 (20)	
2	3 (7)	3 (9)	
PLR			
Normal (≤300)	32 (73)	31 (89)	0.071^a^
High (>300)	12 (27)	4 (11)	

*TNM* tumor-node-metastasis, *5-FU* 5-fluorouracil, *mGPS* modified Glasgow prognostic score, *CEA* carcinoembryonic antigen, *NLR* neutrophil lympocyte ratio, *dNLR* derived neutrophil-to-lymphocyte ratio, *PLR* platelet-to-lymphocyte ratio

^a^Fisher’s exact test

The majority of the patients had a normal CRP (*n* = 43, 54.4%), a normal albumin (*n* = 64, 81%), an mGPS of 0 (*n* = 43, 54.4%), normal hemoglobin (*n* = 45, 57%), normal CEA (*n* = 32, 45%), an NLR of 5 or lower (*n* = 65, 82%), dNLR (*n* = 41, 52%), an NPS of 0 (*n* = 56, 71%), and a PLR of 300 or lower (*n* = 63, 80%) (Table 2).

### Relationships Between Pretreatment Clinicopathologic Factors and Pathologic Response to Treatment

Pathologic response to CRT was significantly associated with pathologic T-stage (*P* < 0.001), TNM stage (*P* = 0.004), vascular invasion (*P* < 0.001), perineural invasion (*P* = 0.045), and radiologic response (*P* = 0.003) (Table 2). Nodal metastases (*P* = 0.189), CEA (*P* = 0.152), and type of chemotherapy (*P* = 0.875) were not associated with pathologic response to treatment (Table 2).

A good pathologic response to treatment was significantly associated with an mGPS of 0 (*P* = 0.022), a normal NLR (*P* = 0.052), and a PLR approaching significance (*P* = 0.071) (Table 2). There was no association between pathologic response to treatment and anemia (*P* = 0.321), dNLR (*P* = 0.705), or NPS (*P* = 0.928) (Table 2).

A logistic regression model using prognostic preoperative factors identified a high mGPS (odds ratio [OR] 0.27; 95% confidence interval [CI] 0.07–0.96; *P* = 0.043) and a poor radiologic response (OR 0.43; 95% CI 0.18–0.99; *P* = 0.048) as strong independent predictors of pathologic response to treatment (Table 3). In the univariate analysis, NLR (OR 0.27; 95% CI 0.08–0.98; *P* = 0.046) was associated with a pathologic response, but not in the multivariate analysis (Table 3). As shown in Table 3, PLR (*P* = 0.057), dNLR (*P* = 0.332), and NPS (*P* = 0.533) were not associated with a pathologic response to treatment. The preoperative logistic regression model was further tested with patients who had nonmetastatic disease only (*n* = 75). The findings showed that mGPS (hazard ratio [HR] 0.25; 95% CI 0.07–0.92; *P* = 0.036) but not radiologic response (HR 0.48; 95% CI 0.20–1.13; *P* = 0.094) remained an independent predictor of response to nCRT.

**Table 3:** Comparison between preoperative clinical factors and good pathologic response to neoadjuvant chemoradiotherapy (nCRT)

	Univariate analysis	*P* value	Multivariate analysis	*P* value
	OR (95% CI)^a^		OR (95% CI)^a^	
mGPS (0 vs 1/2)	0.18 (0.05–0.60)	0.006	0.27 (0.07–0.96)	0.043
NLR (normal/high)	0.27 (0.08–0.98)	0.046	0.72 (0.17–3.04)	0.658
PLR (normal vs high)	0.33 (0.11–1.03)	0.057		
dNLR (normal vs high)	0.73 (0.38–1.39)	0.332		
NPS (normal vs high)	0.77 (0.34–1.75)	0.533		
Radiologic response (good response vs rest)	0.36 (0.18–0.71)	0.003	0.43 (0.18–0.99)	0.048

*OR* odds ratio, *CI* confidence interval, *mGPS* modified Glasgow prognostic score, *NLR* neutrophil lympocyte ratio, *PLR* platelet-to-lymphocyte ratio, *dNLR* derived neutrophil-to-lymphocyte ratio, *NPS* neutrophil-platelet score

^a^Odds ratios and 95% confidence intervals are generated from the logistic regression model

We next created a logistic regression model to test the predictive value of mGPS in the context of prognostic postoperative variables including vascular invasion, perineural invasion, and TNM staging (Table 4). We found that mGPS (OR 0.16; 95% CI 0.04–0.73; *P* = 0.018) remained a strong independent predictor of pathologic response, together with vascular invasion (OR 0.28; 95% CI 0.09–0.84; *P* = 0.023). Radiologic response (OR 0.59; 95% CI 0.22–1.62; *P* = 0.305) was not found to be significant in the model containing pre- and postoperative prognostic clinicopathologic variables.

**Table 4:** Comparison between pre- and postoperative clinicopathologic factors and a good pathologic response to neoadjuvant chemoradiotherapy (nCRT)

	Univariate analysis	*P* value	Multivariate analysis	*P* value
	OR (95% CI)^a^		OR (95% CI)^a^	
mGPS (0 vs 1/2)	0.18 (0.05–0.60)	0.006	0.16 (0.04–0.73)	0.018
NLR (normal/high)	0.27 (0.08–0.98)	0.046	0.98 (0.22–4.39)	0.976
PLR (normal vs high)	0.33 (0.11–1.03)	0.057		
dNLR (normal vs high)	0.73 (0.38–1.39)	0.332		
NPS (normal vs high)	0.77 (0.34–1.75)	0.533		
Radiologic response (good response vs rest)	0.36 (0.18–0.71)	0.003	0.59 (0.22–1.62)	0.305
Vascular invasion	0.20 (0.08–0.52)	0.001	0.28 (0.09–0.84)	0.023
Perineural invasion	0.13 (0.02–1.00)	0.050	0.62 (0.05–7.67)	0.713
TNM stage (1/2 vs 3/4)	0.50 (0.23–1.07)	0.074		
Margin involvement	0.40 (0.13–1.28)	0.121		
Peritoneal invasion	0.25 (0.05–1.18)	0.080		

*OR* odds ratio, *CI* confidence interval, *mGPS* modified Glasgow prognostic score, *NLR* neutrophil lympocyte ratio, *PLR* platelet-to-lymphocyte ratio, *dNLR* derived neutrophil-to-lymphocyte ratio, *NPS* neutrophil-platelet score, *TNM* tumor-node-metastasis

^a^Odds ratios and 95% confidence intervals are generated from the logistic regression model

## Discussion

The study showed an association between a systemic inflammatory response and a poor response to nCRT as determined by the Rödel tumor regression grade for patients undergoing surgery for rectal cancer. This is the first study to assess SIR comprehensively using a variety of prognostic markers of systemic inflammation and its association with response to neoadjuvant therapy in rectal cancer. The findings demonstrated that mGPS is independently associated with response to treatment and may offer further insight into the interaction between the host immune response and tumor regression.^18^,^19^ Our study further demonstrated that response to nCRT, assessed pathologically, is variable (good response of only 44%) and associated with adverse markers of long-term outcome including TNM stage, vascular invasion, and perineural invasion (Tables 1, 2).

Tumor regression grade offers a clinically useful parameter for determining variable rates of response to neoadjuvant therapy. Several scoring systems have been proposed based on the ratio of fibrosis and residual tumor cells, with fairly similar categorical determinants.^11^,^20^,^21^ These scoring systems predict improved disease-free and cancer-specific survival, but it remains to be determined which is best. The main advantage of this method is that it requires no additional laboratory testing or time and has good interobserver concordance. However, it has limitations. Semi-quantitative at best, it is based on selected areas of the tumor and may not fully account for intra-tumoral heterogeneity. Yet, allowing for its limitations, quantifying the ratio of fibrosis to residual cancer tissue is the best method we currently have for predicting response to neoadjuvant therapy. However, this can only be used retrospectively after nCRT and thus cannot be used as a predictive biomarker before surgery.

Whereas findings have shown that pathologic response to nCRT, as measured by TRG, predicts recurrence and survival, the value of post-nCRT MRI remains controversial.^22^ Some studies have previously suggested poor correlation between posttreatment MRI appearances and clinical outcome.^22^–^24^ However, prospectively controlled studies have suggested that tumor regression can be accurately determined using post-nCRT MRI.^25^ A radiologic MRI assessment of tumor regression, as measured by the degree of fibrosis replacing pretreatment tumor, has been shown to correlate with both DFS survival and overall survival for patients with rectal cancer.^25^

Recently, MRI volumetry has allowed more accurate assessment of tumor size than traditional uni-dimensional measurements.^23^ Reports show that MRI volumetry is an accurate predictor of pathologic response to treatment for patients who receive neoadjuvant therapy for rectal cancer.^23^,^26^,^27^ However, the reported studies contained small numbers and did not report correlation with overall survival and DFS.^23^,^26^,^27^ We observed strong associations between radiologic downstaging and pathologic response to nCRT, and thus radiologic response can be a useful adjunct in treatment decisions after nCRT.

In the absence of acute illness, what drives systemic inflammation is poorly understood, but chronic disease, deprivation, and lifestyle changes have been implicated.^28^–^30^ It is likely, however, that in colorectal cancer, systemic inflammation is a response to the tumor and the tumor microenvironment itself. The pro-inflammatory cytokine interleukin-6 (IL-6), appears to play a key role in both local and systemic inflammation in colorectal cancer, with an association between IL-6 expression and more pronounced systemic inflammation (as measured by mGPS) in operable colorectal cancer.^31^,^32^ It has been proposed that elevated serum IL-6 is associated with tumor necrosis, angiogenesis, disease progression, and metastasis.^31^

An elevated CRP has been associated with poor outcome after neoadjuvant therapy and surgery for rectal cancer.^14^,^33^ Kim et al.^33^ observed that CRP expression was significantly higher in nonresponders than in responders, consistent with our findings. We also demonstrated in the univariate analysis that NLR, but not PLR, was associated with response to therapy (Table 3). The prognostic value of NLR and PLR have been validated, and previous studies have demonstrated that both are associated with long-term outcomes for patients undergoing nCRT in rectal cancer.^10^,^34^ We did not find any correlation between dNLR and NPS with response to treatment, which recently has been claimed to be predictive of long-term outcomes in colorectal cancer.^15^,^16^

This study demonstrated that a pretreatment mGPS score of 0 was independently prognostic of a pathologic response to chemoradiotherapy, even when analyzed in a model with prognostic postoperative variables such as TNM stage, vascular invasion, and perineural invasion (Tables 3, 4). Radiologic response assessed after treatment also was closely correlated with a good pathologic response and independently prognostic of such a response in the preoperative regression model (Table 3). This suggests that these preoperative measures can be used to assist in clinical decision making before surgeons proceed with surgery because pathologic response can be assessed only postoperatively. Interdependent postoperative prognostic pathologic variables such as TNM stage, vascular invasion, and perineural invasion are dependent on the pathologic response to nCRT. Thus, measures of SIR were tested in regression models using both pre- and postoperatively available variables to determine its prognostic utility in the pretreatment setting.

Several proposed biomarkers are reported to predict response to neoadjuvant therapy in rectal cancer, yet none have entered routine practice because of problems with methodology and validation.^35^ Serum CRP and albumin, however, are widely used as markers of systemic inflammation. The mGPS is validated as a marker of systemic inflammation and correlates with long-term survival after resection for colorectal carcinoma.^1^

This study was limited by a sample size slightly smaller than those in other published studies of this type.^10^,^33^ This reflects the difficulties of studying a small subtype of patients with colorectal cancer. However, our cohort had comprehensive assessment of the pretreatment systemic inflammatory response, which had not been performed in previous studies. Furthermore, only patients who completed long-course chemoradiotherapy were included in the study because findings have previously shown this to be superior to short-course radiotherapy alone.^36^

Our study did not aim to determine long-term survival and its association with SIR and response to neoadjuvant CRT in this patient cohort. Determinants of survival after resection for rectal cancer are multiple, including postoperative complications, adjuvant therapy, and surgical factors. Thus, our study focused on markers of response to treatment, which have previously been validated as predictors of long-term outcome.^11^,^17^

In conclusion, the current study demonstrated for the first time an association between the presence of a SIR and poor pathologic response to nCRT in patients with rectal cancer. We propose that our study results be validated in an independent cohort with strict follow-up data collection to assess this relationship with long-term survival. The role of anti-inflammatory or immune modulatory therapies in this setting requires further investigation to improve response to nCRT among patients with rectal cancer.
